# Technical-Induced Hemolysis in Patients with Respiratory Failure Supported with Veno-Venous ECMO – Prevalence and Risk Factors

**DOI:** 10.1371/journal.pone.0143527

**Published:** 2015-11-25

**Authors:** Karla Lehle, Alois Philipp, Florian Zeman, Dirk Lunz, Matthias Lubnow, Hans-Peter Wendel, Laszlo Göbölös, Christof Schmid, Thomas Müller

**Affiliations:** 1 Department of Cardiothoracic Surgery, University Medical Center Regensburg, Regensburg, Germany; 2 Department of Internal Medicine II, University Medical Center Regensburg, Regensburg, Germany; 3 Department of Anesthesiology, University Medical Center Regensburg, Regensburg, Germany; 4 Center for Clinical Studies, University Medical Center Regensburg, Regensburg, Germany; 5 Department of Thoracic, Cardiac and Vascular Surgery, University Hospital Tuebingen, Tuebingen, Germany; 6 Department of Cardiothoracic Surgery, Southampton University Hospital Trust, Southampton, United Kingdom; University Hospital Medical Centre, GERMANY

## Abstract

The aim of the study was to explore the prevalence and risk factors for technical-induced hemolysis in adults supported with veno-venous extracorporeal membrane oxygenation (vvECMO) and to analyze the effect of hemolytic episodes on outcome. This was a retrospective, single-center study that included 318 adult patients (Regensburg ECMO Registry, 2009–2014) with acute respiratory failure treated with different modern miniaturized ECMO systems. Free plasma hemoglobin (fHb) was used as indicator for hemolysis. Throughout a cumulative support duration of 4,142 days on ECMO only 1.7% of the fHb levels were above a critical value of 500 mg/l. A grave rise in fHb indicated pumphead thrombosis (n = 8), while acute oxygenator thrombosis (n = 15) did not affect fHb. Replacement of the pumphead normalized fHb within two days. Neither pump or cannula type nor duration on the first system was associated with hemolysis. Multiple trauma, need for kidney replacement therapy, increased daily red blood cell transfusion requirements, and high blood flow (3.0–4.5 L/min) through small-sized cannulas significantly resulted in augmented blood cell trauma. Survivors were characterized by lower peak levels of fHb [90 (60, 142) mg/l] in comparison to non-survivors [148 (91, 256) mg/l, p≤0.001]. In conclusion, marked hemolysis is not common in vvECMO with modern devices. Clinically obvious hemolysis often is caused by pumphead thrombosis. High flow velocity through small cannulas may also cause technical-induced hemolysis. In patients who developed lung failure due to trauma, fHb was elevated independantly of ECMO. In our cohort, the occurance of hemolysis was associated with increased mortality.

## Introduction

The application of extracorporeal membrane oxygenation [ECMO] for patients with refractory respiratory failure failing conventional therapy has increased considerably [1] with improvement on outcome [2–5]. Despite the development of new miniaturized ECMO-systems, technical-induced hemolysis during ECMO therapy remains of concern with a reported incidence between 5 and 18% [6–8]. However, data from clinical practice in adults are scarce and no systematic analysis has been undertaken in a large cohort.

Major contributors of technical-induced hemolysis may consist of sublethal damage to erythrocytes by shear stress [9–11], high extracorporeal blood flow [12], cavitation [13], and pressure changes within the oxygenator [14]. As a result of red blood cell [RBC] destruction, the levels of free plasma hemoglobin [fHb] and lactate dehydrogenase [LDH] can rise significantly during ECMO therapy [15,16]. fHb is cytotoxic resulting in tissue hypoxia and cell death [16,17]. fHb scavenges nitric oxide, leading to inappropriate vasoconstriction, endothelial dysfunction, and platelet aggregation [18,19]. As a consequence, severe complications such as renal dysfunction or multiple organ failure may emerge [20–22]. Therefore, prompt identification of technical-induced hemolysis is essential.

The aim of the current study was to analyze (i) the incidence of technical-induced hemolysis using different modern ECMO-systems, (ii) the specific reasons for episodes of moderate/severe hemolysis, (iii) possible technical causes for hemolysis, and (iv) the influence of high fHb on survival.

## Materials and Methods

### Study population

This is a retrospective analysis on prospectively collected data (Regensburg ECMO database) from 318 consecutive patients on vvECMO (2009–2014) (Table 1). Patients younger than 18 years, with incomplete laboratory records or severe hemolysis (fHb > 500 mg/L) [6,7] prior to ECMO, and those who received ECMO for less than one day were excluded. Ethical approval for publication and need for informed consent was waived by the Ethics Committee of the University of Regensburg, since all devices had been approved for clinical use, no personalized data were used, and only routine laboratory parameters were analyzed.

**Table 1:** Patient data and characteristics before ECMO initiation.

Patients (n)	(n)	318
Age (years)	(years)	52 (38, 61)
Female (n; %)	(n; %)	106; 33
BMI (kg * m^-2^)	(kg * m^-2^)	27.8 (24.6, 33.2)
Ventilation (days)	(days)	2.0 (1.0, 7.0)
SOFA score		11.0 (9.0, 15.0)
LIS		3.33 (3.33, 3.67)
ARF (n; %)	(n; %)	69; 22
norepinephrine (mg/hour)	(mg/hour)	1.5 (0.5, 2.8)
PaO_2_/FiO_2_ (mmHg)	(mmHg)	65 (52, 81)
PaCO_2_ (mmHg)	(mmHg)	63 (51, 80)
apH		7.23 (7.14, 7.33)
TV (mL)	(mL)	469 (392, 560)
TV/kg pred. BW (mL/kg)	(mL/kg)	7.0 (5.8, 8.3)
Minute ventilation (L/min)	(L/min)	10.7 (8.4, 12.6)
PIP (cmH_2_O)	(cmH_2_O)	35 (30, 38)
PEEP (cmH_2_O)	(cmH_2_O)	15 (13, 18)
ECMO indication	Primary lung failure^a^ (n; %)	182; 57
	Secondary lung failure (n; %)	62; 19
	Trauma with ARDS (n; %)	32; 10
	Others^b^ (n; %)	42; 13

Data are median (interquartile range).

SOFA, Sequential Organ Failure Assessment; LIS, Murray lung injury score; apH, arterial pH value; PaCO_2_, partial pressure of arterial carbon dioxide; PaO_2_/FiO_2_, ratio of partial pressure of arterial oxygen and fraction of inspired oxygen; PIP, peak inspiratory pressure; PEEP, positive end-expiratory pressure; TV, tidal volume; BMI, body mass index; ARF, acute renal failure.

^a^ bacterial, viral, fungal, aspiration pneumonia and H1N1 infection.

^b^ other pathologies (eg. pulmonary fibrosis, near drowning, extensive bronchiectasis, pulmonary hemorrhage, tracheal laceration).

### Standard treatment for ECMO patients

Indications for vvECMO treatment have been defined previously [23,24] in accordance with published recommendations [25]. In brief, mechanical ventilation was reduced according to the blood gases (partial pressure of oxygen, PaO_2_ > 70 mmHg, pH normal) aiming for a fraction of inspired oxygen (FiO_2_) < 60%, a peak inspiratory pressure < 28 cmH_2_O, and a tidal volume (TV) < 4 mL/kg predicted bodyweight. The positive end-expiratory pressure (PEEP) initially was kept high to avoid lung de-recruitment ECMO blood flow (maximum 4.5 L/min), FiO_2_ and PEEP were adjusted to maintain an arterial oxygen saturation of > 90%. Sweep gas flow, TV and respiratory rate (RR) were adjusted according to the arterial partial pressure of carbon dioxide (PaCO_2_) aiming at a normal pH. Hemoglobin was kept > 8 g/dL. The anticoagulation protocol was based on a continuous intravenous heparin application. In patients without elevated bleeding risk an activated partial thromboplastin time (APTT) of 50–60 seconds was persued.

### Types of vvECMO-systems

Study patients required one (n = 211) or more oxygenators (n = 107) during the support interval. Different ECMO-systems from three companies (Cardiohelp HLS-set, n = 64; PLS-system, n = 117, Maquet Cardiopulmonary, Rastatt, Germany; Deltastream-system/ILA-activve, n = 85, NovaLung, Heilbronn, Germany; ECC.O5-system, n = 52, Sorin Group, Modena, Italy) were applied as the initial ECMO-system. Variable types of back-flow cannulae (single-lumen, diameter 15, 17, 19, 21 French (Fr); Maquet), drainage cannulae (single-lumen, 21 Fr, 23 Fr; Maquet), and single dual-lumen cannulae (Avalon 23 Fr, 27 Fr, Maquet; NovaPort twin 24 Fr, Novalung) were inserted.

### Continuous veno-venous hemodiafiltration (CVVHD)

CVVHD is a short-term treatment utilized in intensive care unit (ICU) patients with acute or chronic renal failure. Dialysis was undertaken with the aid of the Genius® single-pass batch-dialysis system with a high-flux polysulfone dialyser FX 60 (Fresenius Medical Care, Bad Homburg, Germany). Blood flow and countercurrent dialysate flow ranged between 120 and 250 mL/min. Patients with acute kidney failure requiring ECMO therapy were supported with CVVHD both before initiation or during ECMO treatment.

### Data collection and analysis

Routine laboratory parameters included measurement of LDH and fHb. Blood samples were collected via arterial catheters using lithium-heparin tubes. According to the ELSO Registry, the present study defined hemolysis complicating ECMO support as fHb > 500 mg/L [7, 26, 27]. Significantly elevated values were controlled to verify hemolysis and exclude a sampling error. Prospectively collected clinical, technical and laboratory parameters allowed a retrospective analysis of the prevalence and risk factors for technical-induced hemolysis. Implantation of ECMO-systems in a referring hospital (n = 159) precluded data collection prior to system initiation. Otherwise, for each patient, all the fHb and LDH values were reviewed. Normal subclinical range was defined ≤ 100 mg/L and ≤ 250 U/L, respectively. fHb concentration was determined using a commercial available calorimetric assay (C462-0A Catachem, Oxford, Connecticut). Flow velocity (m/s) within the cannula was defined as the flow rate (m^3^/s) per cross section area of the cannula (m^2^).

Data from all patients were evaluated to give an overview of fHb levels. Then, a detailed case-by-case analysis of outlier peak values of fHb was done to explore individual causes with significant hemolysis. After identifying these episodes, respective data were cleared from further analysis. Finally, all data originating from the first ECMO-run were collected to analyze the effect of different pump and cannula types, duration of the ECMO-run, diagnosis leading to lung failure and ECMO therapy, blood flow, and amount of RBC transfusions per day on the development of hemolysis.

### Statistics

Continous data are expressed as median values and interquartile ranges (IQR: q3-q1); categorical variables are presented as absolute numbers and proportions. Due to the right-skewed distribution of the fHb and LDH values, nonparametric statistical methods were used for all analyses. To analyze the impact of all possible predictors (as listed in Table 2) on fHb and LDH values, linear mixed models based on ranks were used. All predictors were analyzed in univariable models as well as in one multivariable model containing all variables at once. In all mixed models the Kenward-Roger approximation was used, the factor patient was added as a random effect and the correlation structure of the repeated measurements was specified as compound symmetry. Logistic regression models were used to assess the influence of fHb and LDH values on overall mortality and mortality while on ECMO. Odds ratios and corresponding 95% confidence intervals are reported as effect estimates. All reported P values are two-sided, and a P value of 0.05 is considered the threshold of statistical significance. Since the assessment of the predictors was of purely explorative nature, no adjustment for multiple testing was done. All analyses were made using SAS 9.3 (SAS Institute, Cary, NC, USA).

**Table 2:** Association of technical and clinical parameters with fHb and LDH as a marker for hemolysis in vvECMO therapy.

		fHb (mg/L)		LDH (U/L)	
Factors		median (IQR)	p- value	median (IQR)	p- value
Duration, 1^st^ MO (days) ^a^	1st quar. (2 to 8)	56 (36, 92)	-	426 (304, 638)	-
	2nd quar. (8 to 10)	54 (37, 79)	-	476 (348, 692)	-
	3rd quar. (10 to 14)	53 (36, 88)	-	456 (331, 702)	-
	4th quar. (14 to 25)	59 (40, 88)	-	489 (386, 636)	-
Flow velocity (m/s) ^a^	1st quar. (min to 1.81)	51 (36, 80)	-	455 (340, 612)	-
	2nd quar. (1.81 to 2.11)	54 (35, 85)	-	462 (352, 687)	-
	3rd quar. (2.11 to 2.61)	53 (37, 85)	-	473 (334, 663)	-
	4th quar. (2.61 to max)	61 (39, 93)	-	499 (365, 723)	-
RBC (amount per day) ^a^	1st quar. (min to 0.09)	53 (37, 81)	-	489 (355, 703)	-
	2nd quar. (0.09 to 0.27)	50 (34, 73)	-	435 (322, 604)	-
	3rd quar. (0.27 to 0.57)	65 (43, 99)	-	486 (368, 690)	-
	4th quar. (0.57 to max)	62 (37, 104)	-	504 (340, 724)	-
ECMO-system / pump type ^b^	PLS / Rotaflow	60 (38, 102)	-	526 (371, 706)	-
	Hilite 7000/ DP 3	53 (36, 80)	-	430 (337, 624)	-
	ECC.O5 / Revolution	58 (38, 91)	-	461 (332, 668)	-
	Cardiohelp / Rotaflex	52 (35, 78)	-	465 (326, 679)	-
Cannula type / diameter (Fr) ^b^ ^,^ ^c^	Twinport / 24	63 (38, 102)	-	431 (305, 625)	-
	Avalon / 23	57 (34, 96)	-	489 (297, 714)	-
	Avalon / 27	53 (37, 78)	-	474 (390, 622)	-
	single-lumen / 15	51 (33, 90)	-	468 (349, 627)	-
	single-lumen / 17	54 (37, 86)	-	480 (347, 701)	-
	single-lumen / 19	58 (38, 83)	-	439 (340, 616)	-
	single-lumen / 21	44 (35, 66)	-	424 (286, 649)	-
Blood flow (L/min) ^b^	High (≥3.0)	64 (43, 101)	control	503 (372, 725)	-
	Medium (2.6 to 2.9)	53 (36, 81)	0.004	492 (355, 765)	-
	Low (≤2.5)	52 (35, 81)	0.001	446 (330, 618)	-
Indication for ECMO ^b^	Primary lung failure ^d^	54 (36, 83)	≤0.001	462 (339, 676)	0.028
	Secondary lung failure	58 (40, 96)	0.003	562 (407, 716)	control
	Trauma with ARDS	95 (56,129)	control	529 (343, 772)	0.901
	Others ^e^	53 (36, 79)	0.002	432 (321, 599)	0.023
CVVHF ^b^	none	53 (35, 81)	control	443 (321, 629)	control
	During ECMO	57 (38, 89)	0.188	555 (382, 772)	≤0.001
	Before ECMO	63 (41, 102)	0.013	482 (361, 673)	0.005

^a^ p-values refer to the linear mixed model based on ranks with the respective continuous parameter as independent and fHb or LDH as dependent variable. Respective p-values were mentioned in the text.

^b^ p-values refer to the linear mixed model based on ranks with the respective categorical parameter as independent and fHb or LDH as dependent variable (respective p-values were mentioned in the text). Pairwise comparisons (all vs. control) were only performed in case of a significant main effect (p≤0.05).

^c^ Single-lumen backflow (diameter, 15, 17, 19, 21 Fr), dual-lumen cannulae (Avalon 23, 27 Fr) (all Maquet), Twinport 24 Fr (Novalung).

^d^ bacterial, viral, fungal, aspiration pneumonia, H1N1 infection.

^e^ other pathologies (pulmonary fibrosis, pulmonary hypertension, extensive bronchiectasis, pulmonary bleeding, tracheal laceration).

RBC, red blood cells (one RBC contained 300 ml volume); CVVHF, continuous venovenous hemofiltration. fHb, free hemoglobin; LDH, lactate dehydrogenase; Fr, French.

## Results

Patient characteristics and pre-ECMO status of all patients are summarized in Table 1.

### Overview of fHb levels on vvECMO

Including all fHb levels from 318 patients 77.3% were ≤ 100 mg/L, 21.0% were between 100 and 500 mg/L, 1.6% were between 500 and 1000 mg/L, and 0.1% were ≥ 1000 mg/L [27].

### Identification of specific episodes of moderate/severe hemolysis

**Fig 1:**
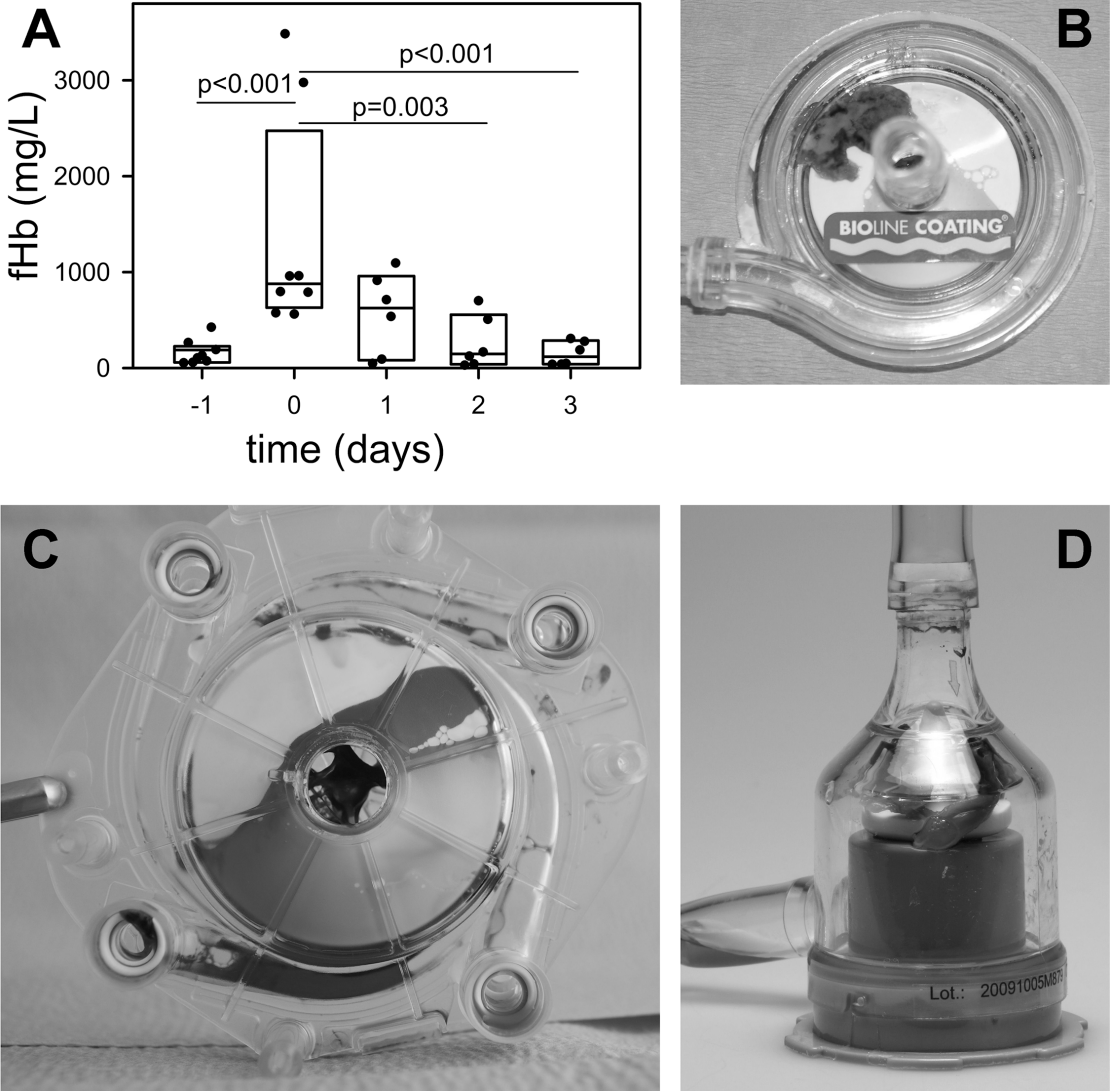
Pumphead thrombosis was documented in 8 patients. (A) Within 1 day free hemoglobin (fHb) increased significantly. After removal of the system (day 0) fHb normalized within 2 days. Data are presented as median (25/75 percentiles). Data points indicate individual patient data. Clots within pump heads of a Rotaflow (B), Rotaflex (C) and Deltastream DP3 (D).

A sudden pronounced increase in fHb levels (≥ 1000 mg/L) can indicate pumphead thrombosis (PHT) [6]. In 8 patients fHb levels increased markedly within 24 hours (Fig 1A, p≤0.001), and normalized within 2 days after pumphead replacement. Clots were observed inside the pumpheads (PLS, n = 3; Cardiohelp, n = 3; Deltastream DP3, n = 2) (Fig 1B–1D). Furthermore, pre-existing (before ECMO initiation) moderate elevations of fHb were seen due to sepsis (n = 4), multiple trauma (n = 4), and cardiac surgery (n = 3). Also, coagulation disorder (sudden increase in D-dimers, increase in pressure drop across the MO, n = 2) and fulminant pulmonary embolism (n = 1) during ECMO support resulted in high fHb levels. As these episodes were explained by clearly distinguishable triggers, they were excluded from further analysis which investigated technical-induced hemolysis.

In contrast, acute clot formation within the oxygenator (AOT, acute oxygenator thrombosis; n = 15) resulted in a marked increase in pressure-drop across the oxygenator, but fHb remained stable (before replacement, 62 (39, 148) mg/L; day of replacement, 86 (69, 113) mg/L).

### Identification of potential technical and clinical risk factors for hemolysis in vvECMO therapy

Data from the first ECMO run were analysed to evaluate the effect of ECMO duration, pump and cannula type, blood flow, flow velocity, diagnosis resulting in ECMO treatment, concurrent use of CVVHD, and the impact of RBC transfusions on fHb. The association of potentially relevant technical and clinical factors with hemolysis during vvECMO therapy is summarized in Table 2.

**Fig 2:**
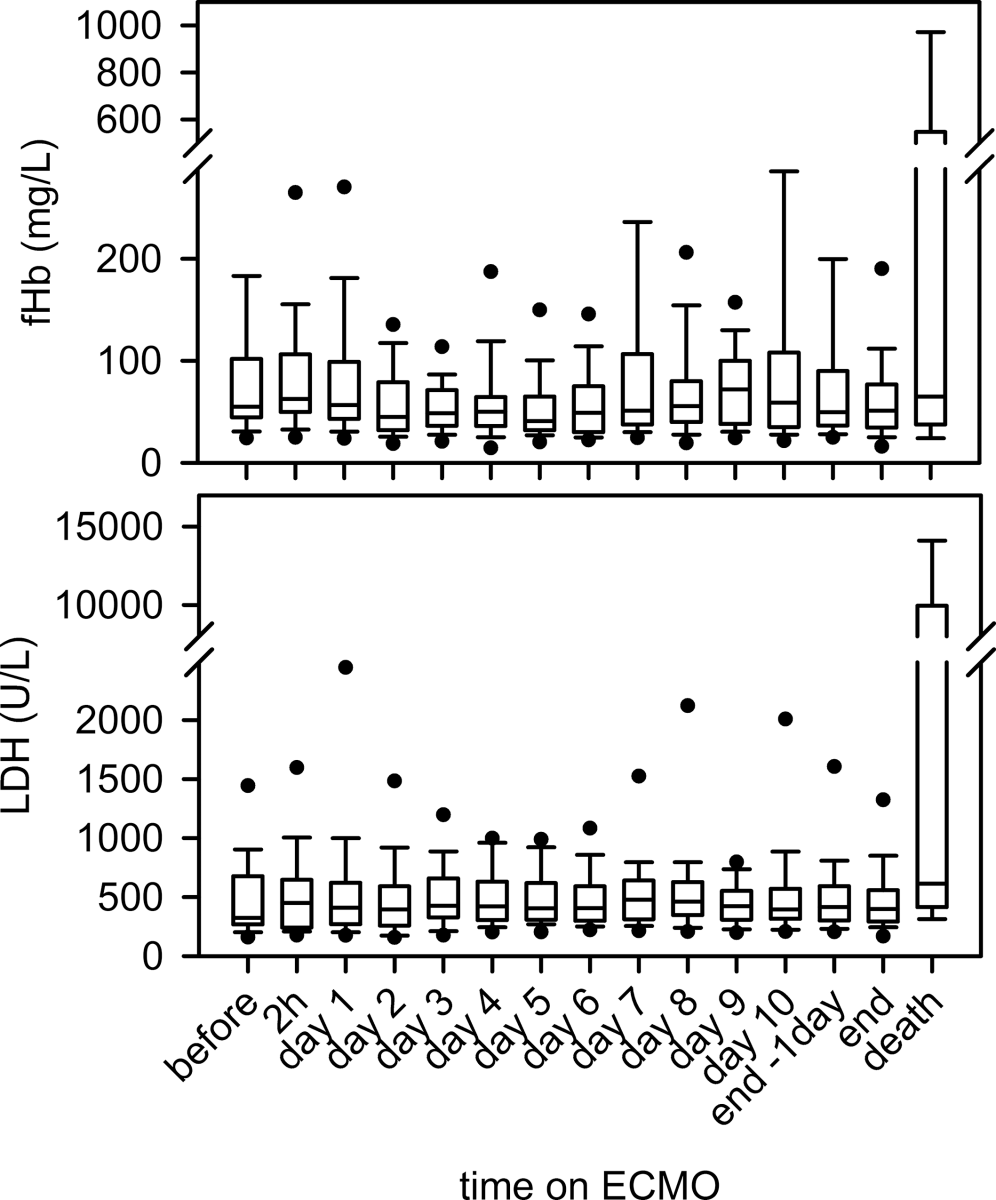
Time course of fHb and LDH on vvECMO. Neither fHb nor LDH levels changed after initiation of vvECMO. Termination with successful weaning (end) did not change fHb and LDH levels. Only patients that died on the system (death) showed a significant increase in fHb and LDH values (p = 0.001, each).

Regarding the time course on the first MO (Table 2), no significant link between was found for fHb (p = 0.363) or LDH (p = 0.287). In a data set of 65 patients with paired documentation of fHb values before, 2 hours and daily after implantation, there was no alteration in the fHb levels (including end of therapy) (Fig 2). The total ECMO duration of the first MO did not impact fHb (p = 0.095) and LDH (p = 0.134) values either.

#### ECMO-system/blood pump and cannulae

**Fig 3:**
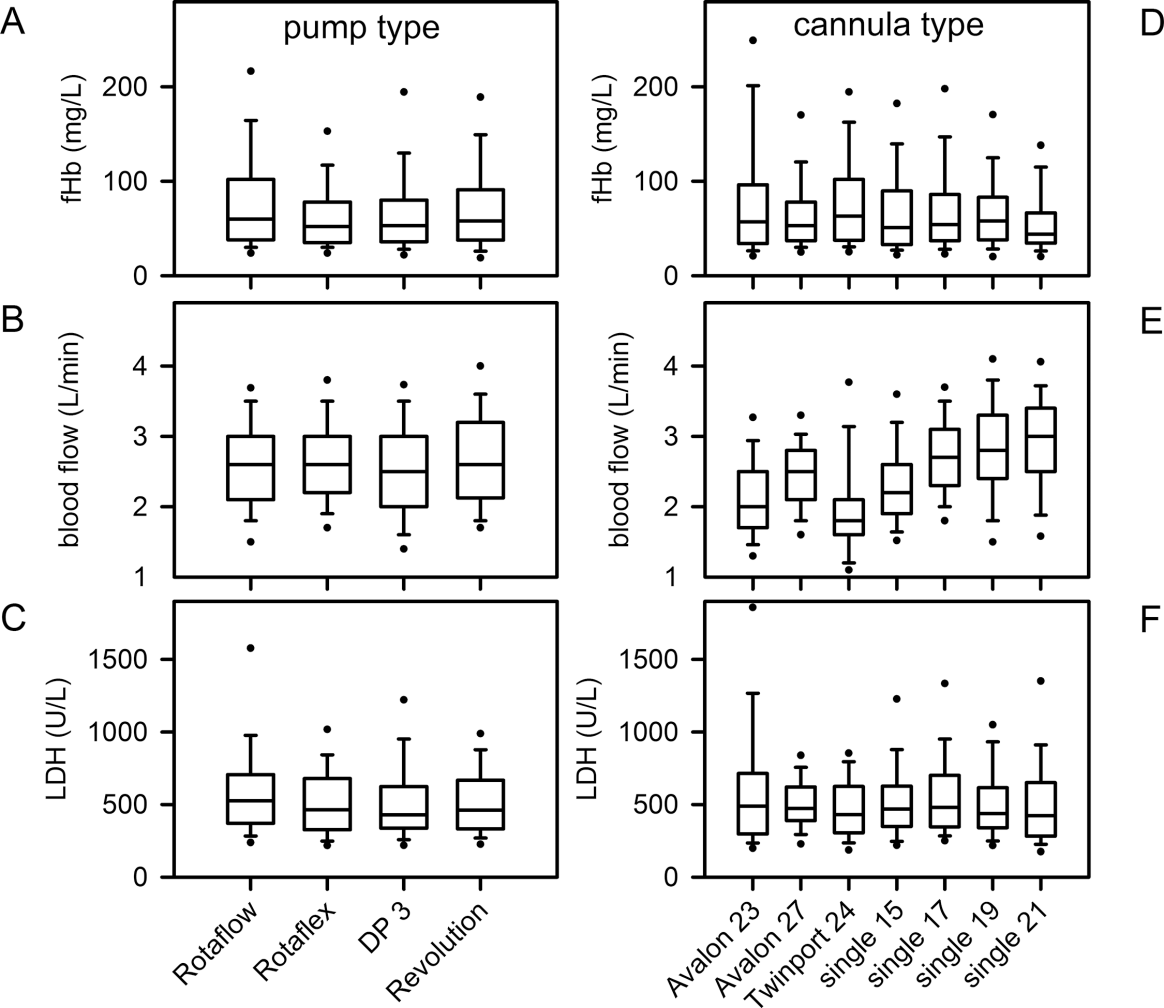
Effect of pump type and cannula type on fHb, chosen blood flow and LDH during ECMO therapy. Data are presented as median (interquartile range), error bars are 5/95 percentiles, circles are extreme values.

The blood pumps of the different ECMO-systems were operated at distinct device-settings [28]. Due to alternative technical design, the DP3 was run on signifcantly higher rotational speed. Nevertheless, fHb values were independent of the pump type (data not shown). Neither pump type (Fig 3A–3C), nor cannula type (Fig 3D–3F) induced an increase in fHb (pump, p = 0.242; cannula, p = 0.906) or LDH levels (pump, p = 0.180; cannula, p = 0.262) on ECMO within the applied blood-flows. However, the individual cannula size required adaptation of blood flow (Fig 3E). At higher blood flow (≥3.0 L/min), the NovaPort twin 24 Fr cannula induced significantly higher fHb levels (116 (94, 129) mg/L compared to Avalon 27 Fr (61 (40, 83) mg/L); single 17 Fr (63 (43, 96) mg/L); single 19 Fr (62 (40, 99) mg/L); single 21 Fr 53 (39, 73) mg/L); each, p≤0.001). Considering the flow velocity within cannulae, fHb increased slightly at higher flow velocity (p = 0.003), while LDH was not changed (p = 0.090) (Table 2). Due to high variance in the data, it was not possible to define a cut-off value for flow velocity for individual cannulae on the basis of fHb.

#### Extracorporeal blood flow

**Fig 4:**
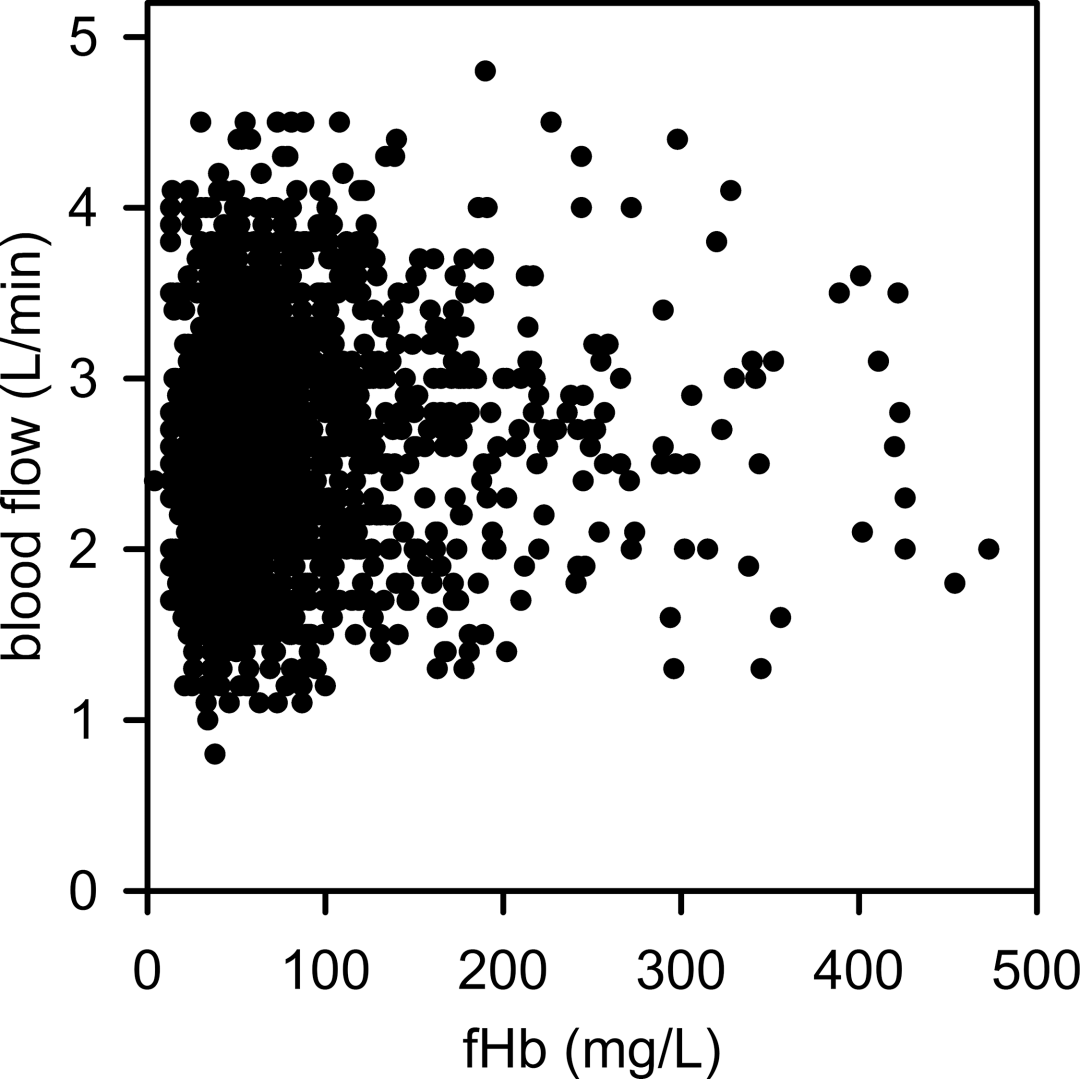
Correlation of fHb levels and extracorporeal blood flow on vv-ECMO failed. More than 70% of the fHb levels were below 100 mg/L.

Blood flow was classified into 3 groups [low (≤ 2.5 L/min), medium (2.5–3.0 L/min), high (≥ 3.0 L/min)]. The linear mixed models demonstrated a significant impact of blood flow on fHb (p = 0.002), but not on LDH (p = 0.134). Low and medium blood flows were associated with a lower fHb level, while higher blood flow resulted in a moderate increase in fHb (high vs. low, p = 0.004; high vs medium, p = 0.011; Table 2), but blood flow between 3 and 4.5 L/min per se did not cause extensive hemolysis, as about 75% of all fHb levels were below 100 mg/L (Fig 4).

#### Effect of negative pressure

fHb levels in patients perfused with Avalon 27 Fr and single-lumen Maquet 21 and 23 Fr drainage cannulae were analyzed for the hemolytic effect of negative pressure on blood trauma. Including all data sets, single-lumen cannulae were operated on average of a higher blood flow (single 21 Fr, 2.6 (2.0, 2.9) L/min, p = 0.02; single 23 Fr, 2.7 (2.2, 3.0) L/min, p = 0.005; vs Avalon 27 Fr, 2.4 (2.1, 3.0) L/min) and generated less negative pressure (single 21 Fr, -10.5 (-28, 1.0) mmHg, p≤0.001; single 23 Fr, -7.0 (-18.0, 4.0) mmHg, p = 0.005; vs Avalon 27 Fr, -29.0 (-56, -4) mmHg). Within the used blood flows, there was no difference in fHb levels (single 21 Fr, 53 (38, 78) mg/L; single 23 Fr, 59 (37, 84) mg/l; vs Avalon 27 Fr, 57 (30, 94) mg/L); non-significant) or LDH (not shown).

#### Diagnostic groups and hemolysis

**Fig 5:**
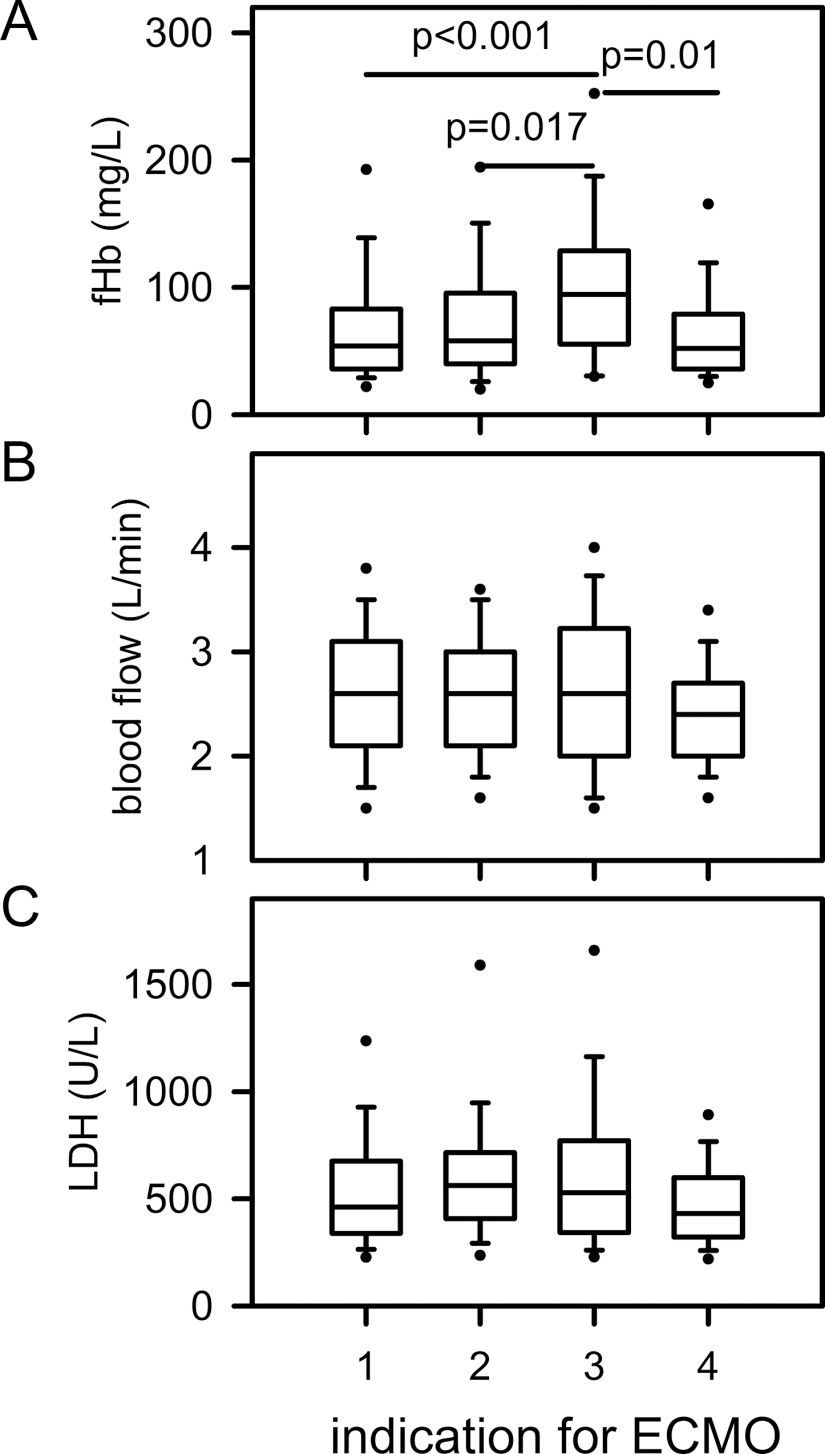
Diagnostic group and fHb, chosen blood flow and LDH. Patients requiring ECMO therapy due to ARDS triggered by trauma (#3, n = 32) presented significantly higher fHb levels. Data are presented as median (25/75 percentiles), Error bars are 5/95 percentiles, circles are extreme values. ECMO indications: #1, primary lung failure (bacterial, viral, fungal, aspiration pneumonia, H1N1 infection); #2, sepsis with secondary lung failure; #3, trauma with ARDS; #4 other pathologies (eg. Pulmonary fibrosis, near drowning, extensive bronchiectasis, pulmonary hemorrhage, tracheal laceration).

To analyze the influence of different causes resulting in lung failure and ECMO therapy on fHb and LDH, patients were categorized into 4 diagnostic groups (Table 2, Fig 5): lung failure primarily due to pulmonary causes (group #1), due to extrapulmonary sepsis (group #2), due to trauma (group #3) and miscellaneous causes (group #4). The linear mixed models showed a marked effect of diagnostic groups on fHb (p≤0.001). Trauma patients (#3) had significantly higher fHb levels (vs. #1: p<0.001, vs #2: p = 0.017, vs #4: p = 0.010) (Fig 5A). LDH showed a similar trend (p = 0.046), but no significant difference in the pairwise comparisons was found in the multiplicity adjusted p-values (Fig 5C).

#### Renal failure and continuous vv hemodiafiltration

Renal failure and the need for CVVHD resulted in an increase of fHb (p = 0.036) and LDH (p≤0.001) compared to patients without renal failure. ECMO therapy without CVVHD was associated with lower levels of fHb and LDH compared to patients with CVVHD-requirement before and/or during ECMO therapy (no CVVHD vs CVVHD before ECMO, p = 0.034 for fHb, p = 0.014 for LDH; no CVVHD vs CVVHD during ECMO, p≤0.001 for LDH, non significant for fHb).

#### RBC transfusion

An increased demand of RBCs per day was associated with a rise in fHb (p = 0.001) (Table 2). The large variance of the data prevented further detailed analysis of causes. There was no effect on LDH (p = 0.107) observed, probably due to an already markedly elevated level of LDH in these critically ill patients, so that an additional increase due to hemolysis could not be elaborated.

### Multivariable models

To determine the independent effect of each factor, the variables diagnostic group, number of RBCs per day, flow velocity, blood pump type and CVVHD were included into the analysis. The multivariable model showed, that higher levels of fHb were significantly associated with patients who required ECMO therapy due to acute respiratory failure triggered by multiple trauma (p = 0.003), high numbers of RBCs per day (p = 0.017) and high flow velocity (p = 0.013). Blood pump type and need of CVVHD had no independent effect on fHb values (p = 0.670, p = 0.305).

### Outcome

**Fig 6:**
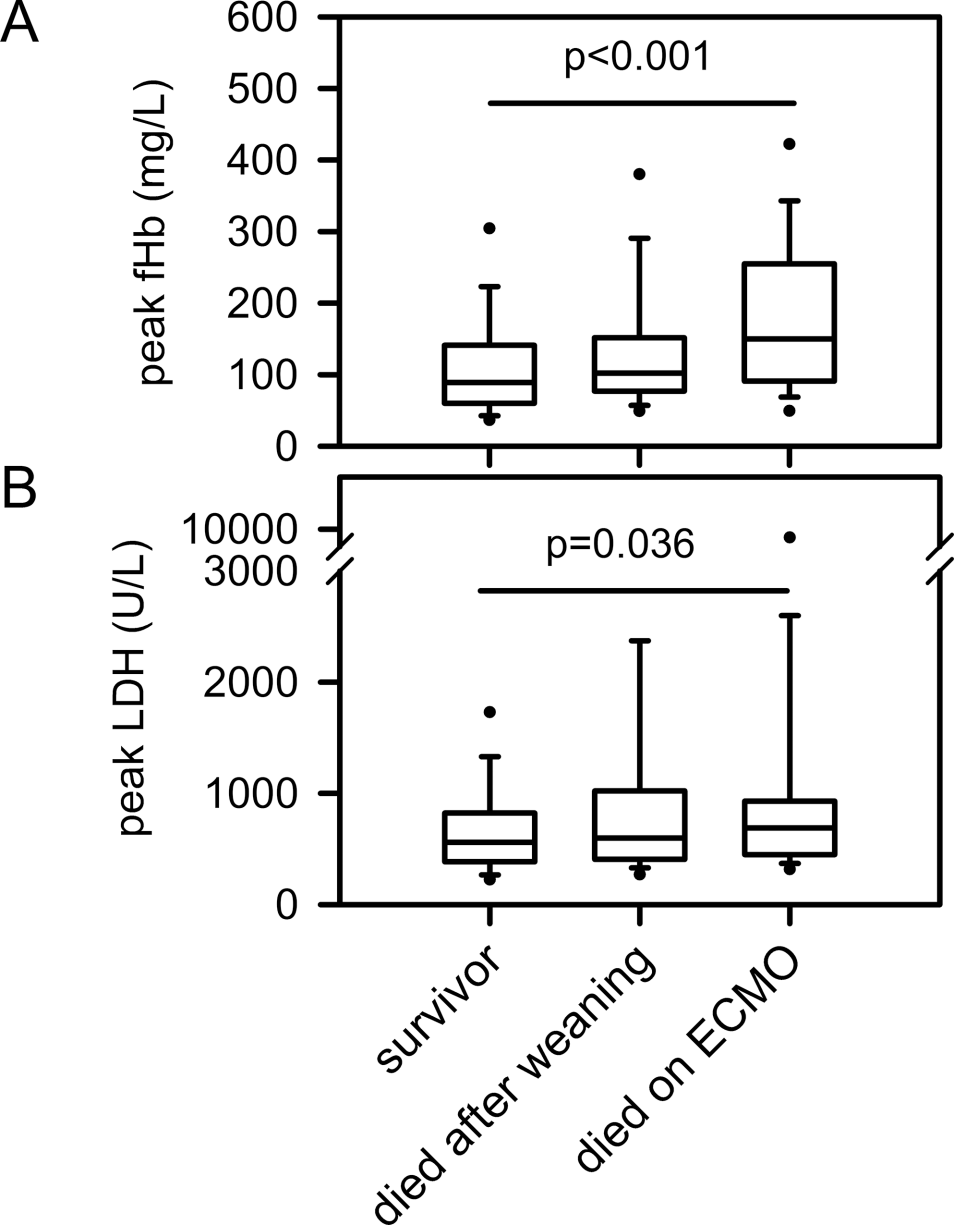
Mortality on ECMO and hemolysis. Data are presented as median (interquartile range), error bars are 5/95 percentiles, circles are extreme values.

Peak levels of fHb and LDH were obtained from each patient to describe their impact on survival. Mortality on ECMO was both linked to higher peak levels of fHb and LDH (Fig 6). Logistic regression models demonstrated that high peak levels of fHb and LDH were associated with an increased risk of overall mortality (odds ratio (per 10 mg/L fHb increase): 1.054 (95%-CI, 1.028–1.080), fHb, p<0.001; LDH, p = 0.036) and an increased risk for mortality on ECMO (odds ratio (per 10 mg/L fHb increase): 1.059 (95%-CI, 1.032–1.087), p≤0.001).

## Discussion

Besides thromb-embolic complications and bleeding, hemolysis was a serious problem with early extracorporeal lung support. In recent years several new, technically optimized ECMO-systems have been developed. Improved pump design, low resistance oxygenators, plasma-tight gas exchange membranes made of poly-methyl-pentene and surface-coating of all compartments resulted in less need of systemic anticoagulation and better biocompatibility also in mid- and long-term application. To our knowledge, there has been no comprehensive analysis on the incidence and the causes of hemolysis in the adult population with this new devices.

Recently, it was shown that only one third of pediatric patients on ECMO were free of hemolysis, but almost 7% displayed severe hemolysis with fHb values larger than 1000 mg/L [27]. Hemolysis was observed commonly and associated with a number of adverse outcomes. In the present study, severe hemolysis was seen in 8 patients (2.5%) and always indicated PHT. In consequence, a rapid increase in fHb in a four-digit range should immediatelly raise suspicion that the pumphead may be partially clotted and may have to be exchanged. PHT may cause a noisy pump run, but may be undetectable by inspection alone. A rotating clot in the pumphead will cause excessive shear forces and mechanical destruction of erythrocytes. Exchange of the pumphead will often normalize fHb within two days [6,29]. If platelets fall in parallel, a heparin-induced thrombocytopenia must be ruled out; a change of anticoagulation is advisable until receipt of laboratory values.

In contrast, AOT or clotting within the oxygenator did increase D-dimers, but not fHb, probably because no disruption of erythrocytes occurred [30,31].

Of a total of 4,142 measurements of fHb, only 1.7% were above 500 mg/l. This striking difference to the pediatric population may be explained by the fact, that we solely analysed vvECMO runs, whereas Lou et al. [27] mainly investigated pediatric and neonatal patients on veno-arterial ECMO. In addition, the coagulation system and the molecular structure of RBCs of neonates is differing, which may result in a higher incidence of hemolysis [27].

Due to the risk of hemolysis on ECMO support [8], daily routine fHb monitoring has been recommended for both pediatric [27,32] and adult patients [6]. Hemolysis has several detrimental effects as described above. In this context, it was shown that fHb-derived free iron exposure may lead to direct kidney damage as a consequence of tubular obstruction and through the generation of reactive oxygen pathway [16,21]. Thus, regular monitoring is important, and the reasons for hemolysis have to be elucidated.

Technical-induced hemolysis can result from several factors [7] including the circuit components (oxygenator, pump, cannula) as well as the pre-ECMO patient characteristics (e.g. reason of acute lung failure, requirement of CVVHD, RBC transfusion, age of RBC) [33].

In previous bench studies, a correlation between hemolysis and the pressure drop across the oxygenator during ECMO was proven for polypropylen oxygenators that were available for pediatric cardiopulmonary bypass [34]. The use of a smaller dimension pediatric oxygenator compared to an adult oxygenator resulted in greater hemolysis and a higher pressure gradient, which may generate higher shear forces [14]. In the present study, which excluded patients requiring pediatric oxygenators, none of the applied oxygenators per se induced hemolysis in clinical practice. Within the used blood flow, this was true for the ECC.O5 as well, even though this oxygenator has a smaller surface area and a higher trans-oxygenator pressure gradient [28]. Comparable results were reported by Palanzo et al. [35], who did not observe differences in hemolysis in different pump types. In contrast, Klaus et al. [36] demostrated that high rotational speed at physiological flow may lead to high fluid stress in some pump regions resulting in blood damage. We could demonstrate that the Deltastream system, which runs approximately on double speed compared to the other pump types, does not provoke more hemolysis at identical blood flow; thus, rotational speed per se does not cause hemolysis. However, we saw fHb levels slightly, but significantly increased at flow rates between 3.0 and 4.5 L/min. Theoretically, long-term blood flow rates ≥ 3.0 L/min can result in the induction of subhemolytic trauma of erythrocytes [10,11], which may lead to changes in cell geometry, shortening of cell life span, and Hb leakage, albeit without complete rupture of RBCs [37]. In contrast, usage of modern ECMO systems did not increase fHb over time.

Higher levels of fHb may be due to higher blood flows, but could also be explained by a higher severity of disease with sepsis. Therefore, we analysed whether an increase of flow velocity causes more hemolysis. We could demonstrate a correlation between the two factors especially with higher flow velocities (Table 2). Within prespecified blood flows no increase of hemolysis occurred, but an excessive blood flow in relatively small cannulae can result in marked hemolysis. In particular, this was proven for the NovaLung Twinport 24 Fr, which was designed for CO_2_-removal on blood flows up to 2 l/min and can cause hemolysis if run on higher flows. Therefore, both the choice of cannula and adaptation of the blood flow can prevent technical-induced hemolysis [38].

It is well known that high negative pressures, e.g. generated by kinking of lines or obstruction of in-flow can cause cavitation and hemolysis [7]. The negative pressures may intermittently reach -700 mmHg for a short term, which will not be recorded by routine measurement. Therefore, from our data it was impossible to describe the direct effects of excessive negative pressures on hemolysis. 27 Fr Avalon double-lumen cannulae generated a higher negative pressure on average, but within the used blood flows this was not mirrored in an increase of fHb.

Patients, who required CVVHD prior to ECMO initiation displayed a significantly higher level of fHb. Comparable results were demonstrated in pediatric patients requiring ECMO support following congenital surgical repair [22]. It was speculated that sublethal erythrocyte damage in the ECMO circuit was followed by frank hemolysis of the pre-damaged erythrocytes in the dialysis circuit [10,22]. To substantiate this assumption, further investigation is needed, as severity of disease again may be the reason for higher fHb in this patient group.

Patients who developed lung failure after multiple trauma showed significantly higher fHb values compared to patients with lung failure of other diagnostic groups. These patients often required ECMO support after severe hemorrhage, high velocity accidents or blast injury, extensive surgery and necessity for multiple transfusions [39]. Both increased risk of hemorrhage and requirement for RBC transfusion has been reported to be accompanied by higher levels of circulating fHb [40]. This may either be due to release of fHb from extensive hematoma or faster destruction of transfused erythrocytes, esp. if stored for prolonged periods [41].

Multivariable statistical analysis revealed that higher levels of fHb are associated with multiple trauma, increased RBC transfusion and high flow velocity. Increased levels of fHb and LDH were associated with a higher mortality in our study, which is in accordance with previous data on patients with sepsis [42]. Due to the retrospective character of our study, it has to be pointed out that hereby a causal relationship is not proven.

Our study has several limitations. It is a retrospective monocentric analysis which intended to find possible causes for hemolysis in adult patients on vvECMO. We have tried to study outliers individually to identify rare causes of hemolysis, as statistical methods are inclined not to detect the extremes. Almost all patients who were in need of ECMO had severe sepsis, which itself can cause hemolysis, thus, the observed levels of fHb most likely were not solely due to ECMO, but may have been related to other causes. Our investigations relate to the blood flows and cannulae used in this study and can not be generalized to other centers which may use larger blood flows or smaller cannulae. Finally, intravascular hemolysis and increased levels of fHb are a late marker of blood trauma. Activation and destruction of platelets may be a more suitable parameter to detect subtle injury.

## Conclusions

This is the first study with a large cohort of adult patients treated with vvECMO which investigated the incidence and possible causes of hemolysis. It proves that modern miniaturized ECMO devices are safe and generally do not cause significant hemolysis during long-term use. LDH commonly is elevated in patients with severe sepsis and thus is an unreliable parameter for hemolysis on ECMO. Marked hemolysis is seen in about 3% of patients and often due to a clot within the pump head, which should trigger prompt exchange. Expectedly, high flow velocity can cause more blood damage. In consequence, the size of cannulae has to be guided by expected need of blood flow. It is advisable that fHb is routinely measured in all patients on ECMO on a daily basis to detect problems at an early stage.
